# Supplementary material for: Purification and Identification of Flavonoid Molecules from Rosa setate x Rosa rugosa Waste Extracts and Evaluation of Antioxidant, Antiproliferative and Antimicrobial Activities
Source: Molecules. 2022 Jul 8;27(14):4379. doi: 10.3390/molecules27144379 (PMC9323010; doi:10.3390/molecules27144379)
Supplement: Supplementary file 1 [file molecules-27-04379-s001.zip › molecules-1781680-supplementary.pdf]

Supplementary material

Figure. S1. Effects of ethanol concentrations on the flavonoids purity during HP20 resin column chromatographic

purification.

Figure. S2.-S15. Targeted-MS/MS spectra of 14 flavonoids detected in the purified extracts from *Rosa setate x Rosa rugosa* waste.

Table S1. Recovery rate and purity of the flavonoids in *Rosa setate x Rosa rugosa* wastes with different MARs.

Table S2. Physical properties and manufacturer of eleven types of macroporous adsorption resins.

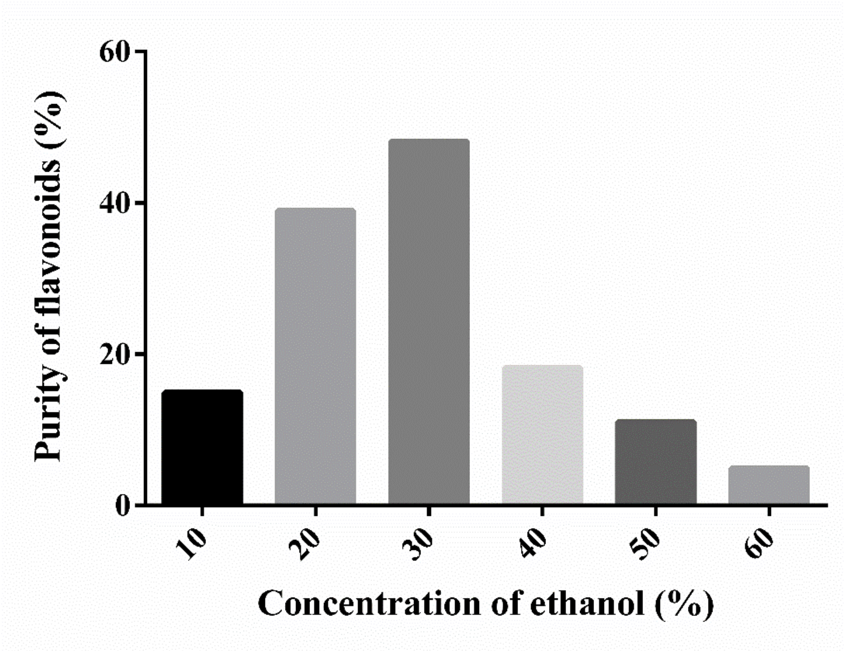

Figure S1. Effects of ethanol concentrations on the flavonoids purity during HP20 resin column chromatographic purification.

Figure S2. The MS-MS spectrum of peak 1 (Quercetin-3,4'-O-diglucoside).

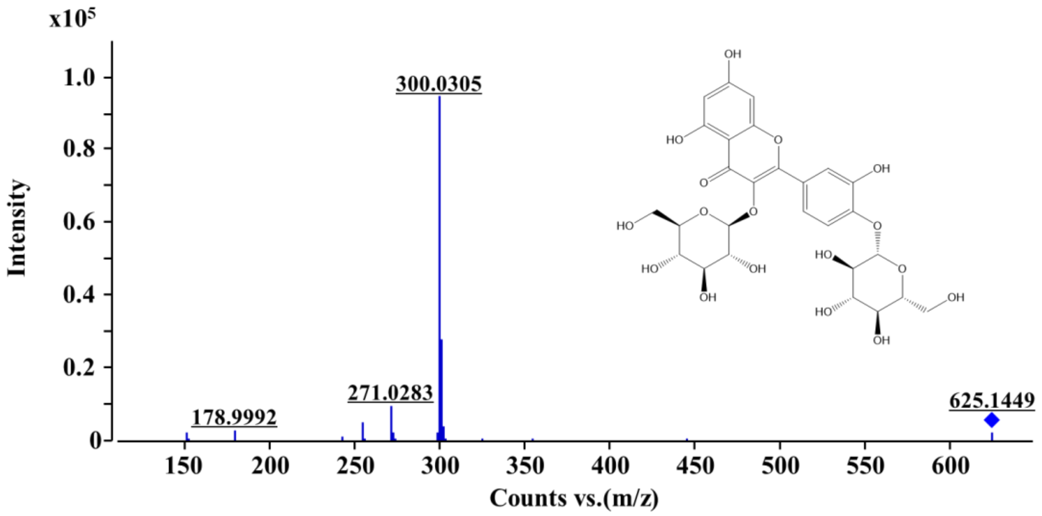

Figure S3. The MS-MS spectrum of peak 2 (Quercetin-3-O-sophoroside).

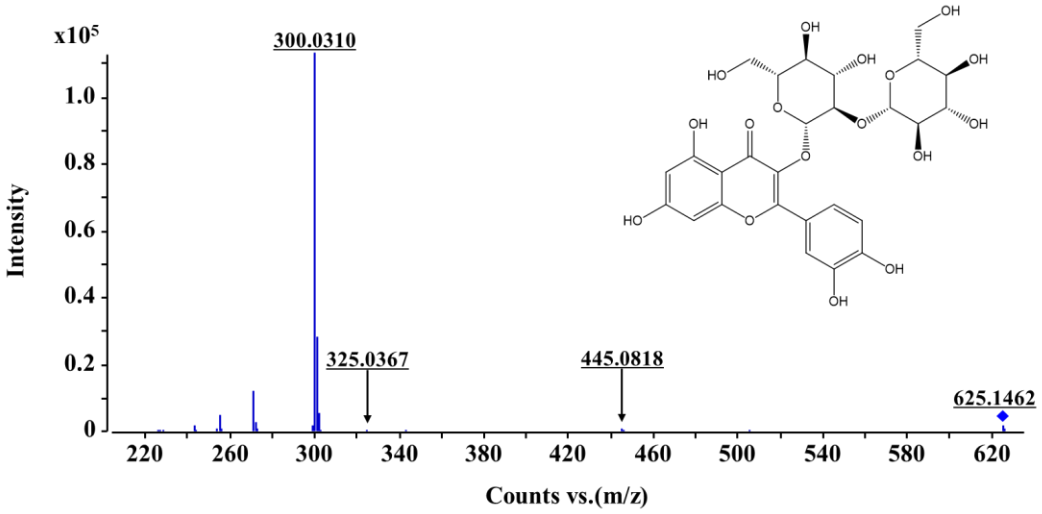

Figure S4. The MS-MS spectrum of peak 3 (Kaempferol-3,4'-di-O-glucoside).

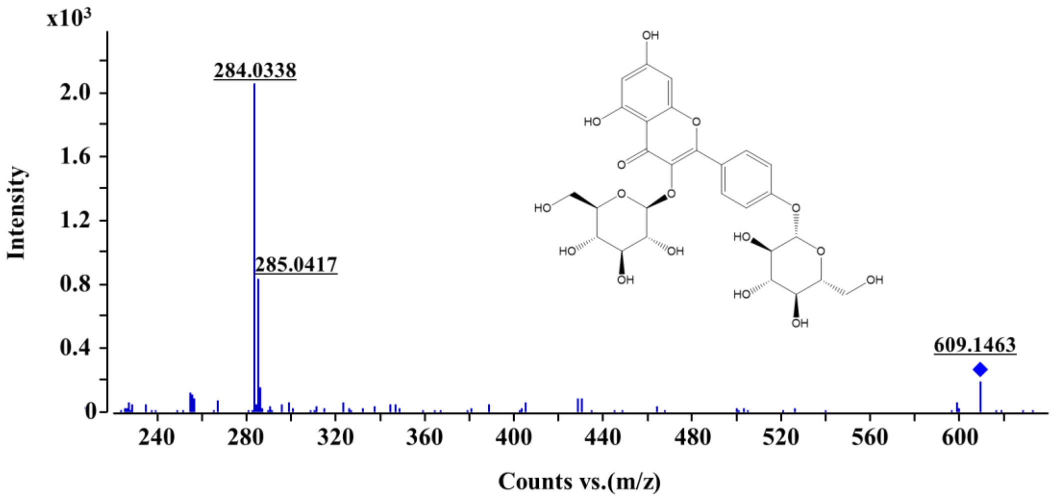

Figure S5. The MS-MS spectrum of peak 4 (Isorhamnetin-3-O-sophoroside)

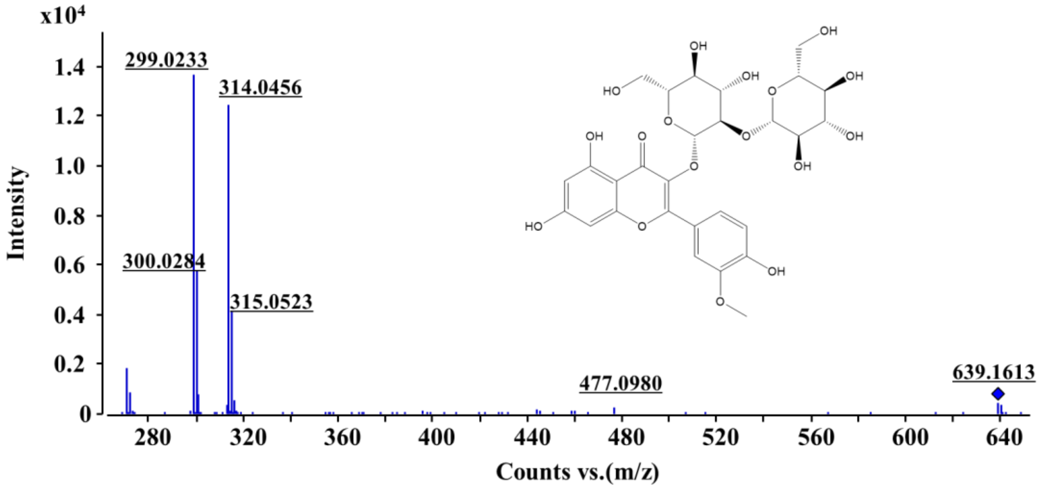

Figure S6. The MS-MS spectrum of peak 5 (Kaempferol-3-O-sophoroside).

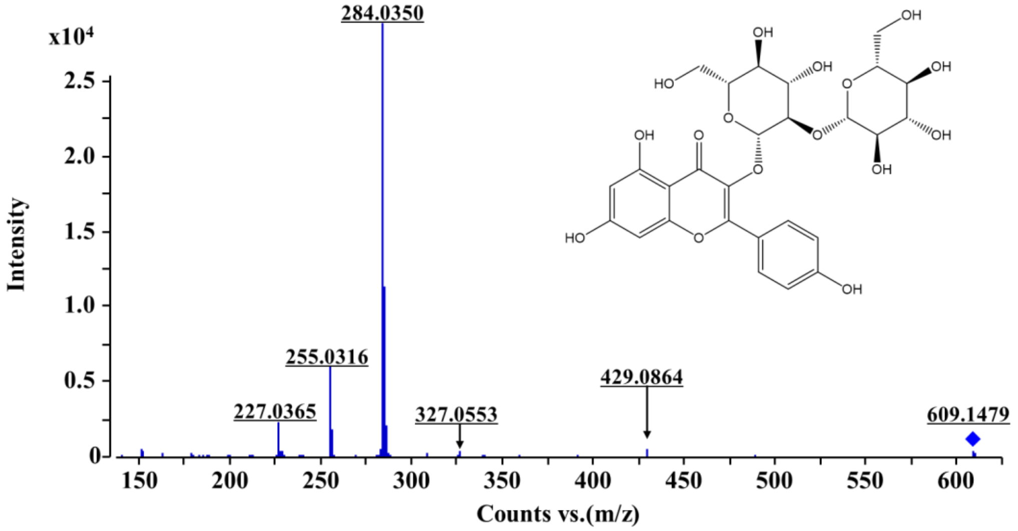

Figure S7. The MS-MS spectrum of peak 6 (Quercetin-3-O-galactoside (hyperoside)).

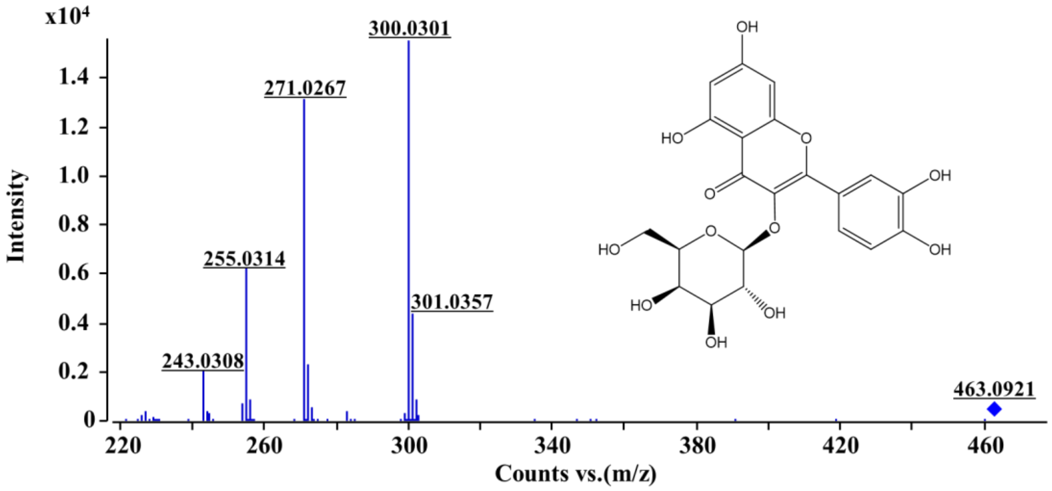

Figure S8. The MS-MS spectrum of peak 7 (Quercetin-3-O-rhamnopyranosyl-(1→6)-glucopyranoside (rutin)).

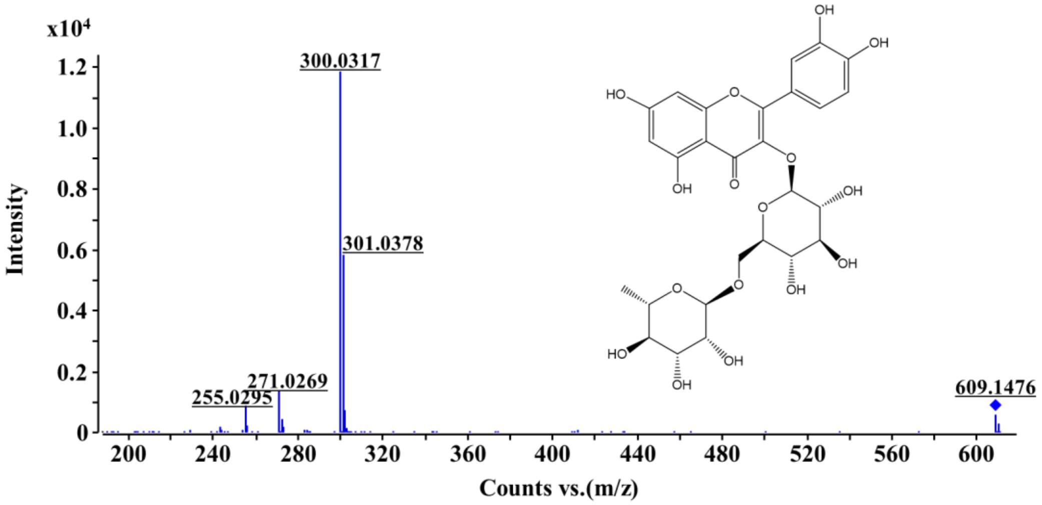

Figure S9. The MS-MS spectrum of peak 8 (Quercetin 3-O-glucoside (isoquercitrin)).

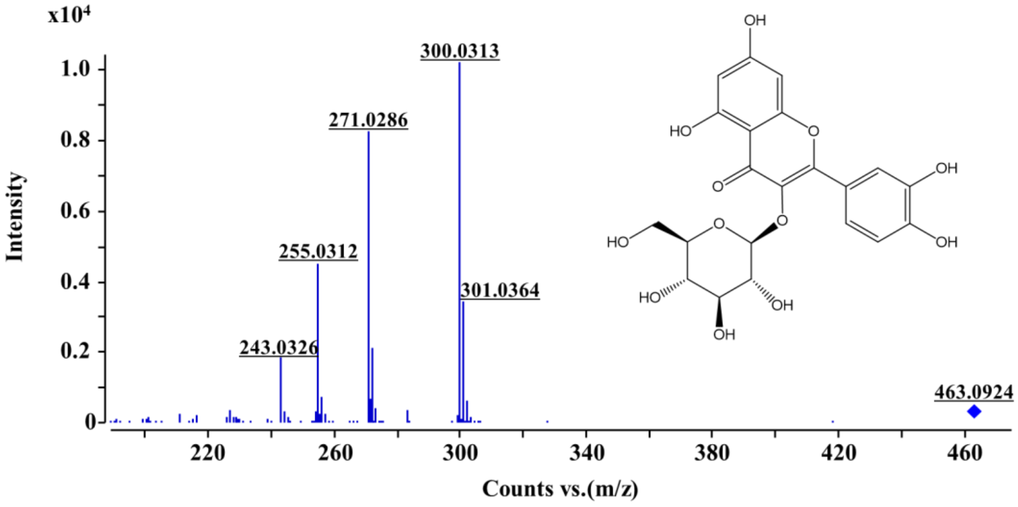

Figure S10. The MS-MS spectrum of peak 9 (Quercetin-3-O-pentoside isomer1).

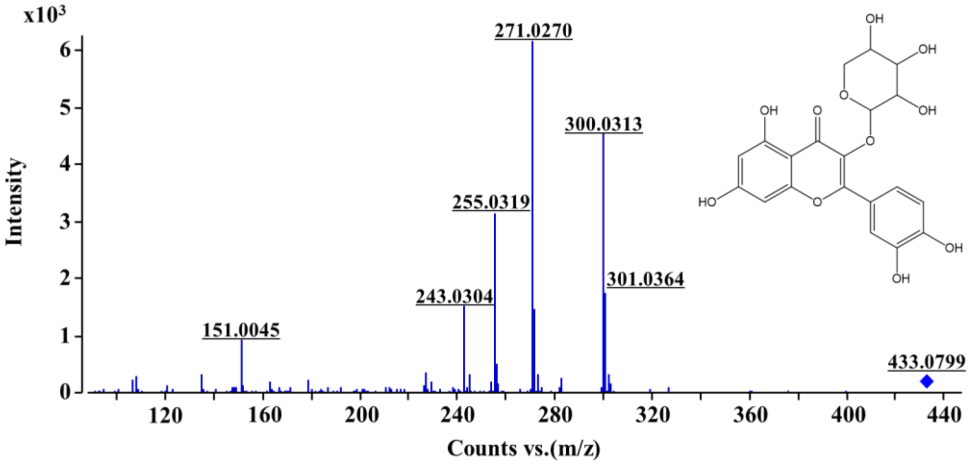

Figure S11. The MS-MS spectrum of peak 10 (Kampferol-3-O-(6''-galloyl)-β-D-glucoside).

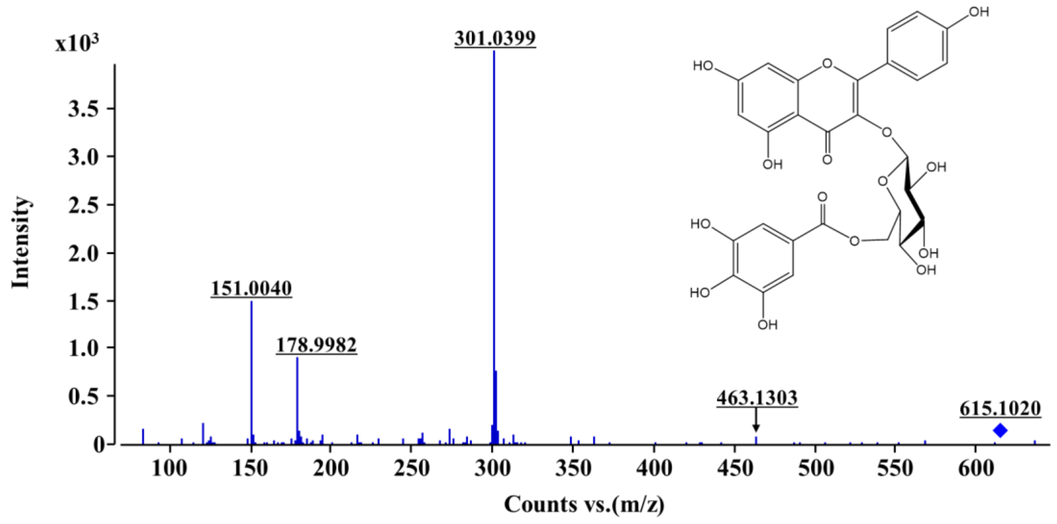

Figure S12. The MS-MS spectrum of peak 11 (Kaempferol-3,7-di-O-rhamnoside).

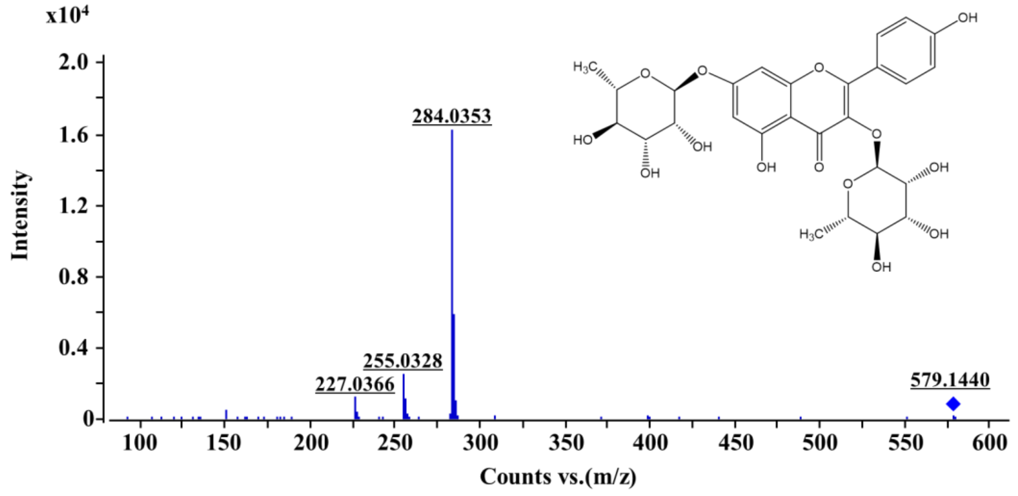

Figure S13. The MS-MS spectrum of peak 12 (Quercetin-3-O-pentoside isomer2).

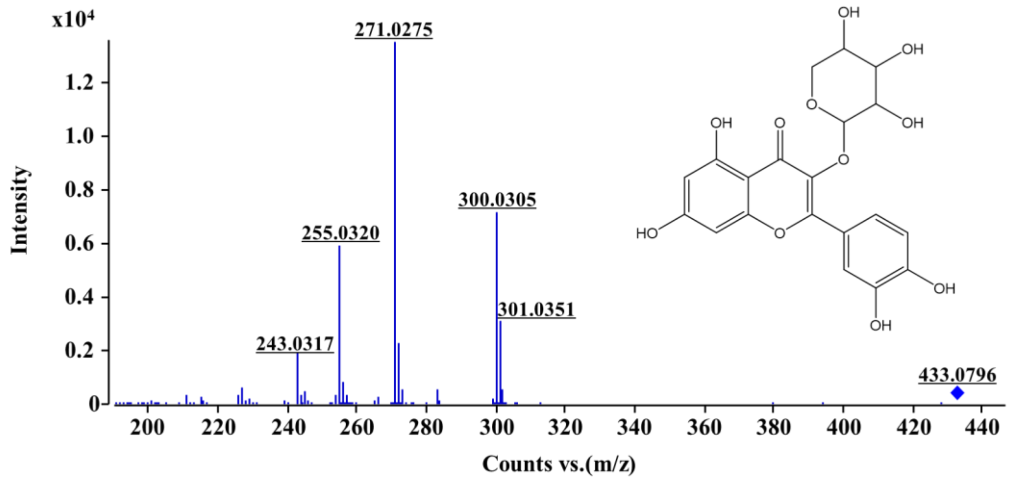

Figure S14. The MS-MS spectrum of peak 13 (Kaempferol-3-O-glucoside (astragalin)).

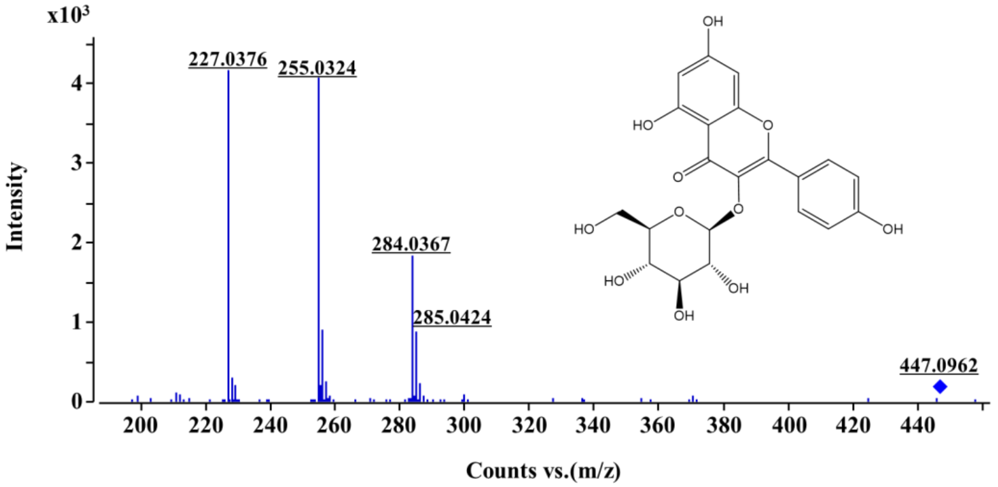

Figure. S15. The MS-MS spectrum of peak 14 (Quercitrin-3-O-rhamnoside (quercitrin)).

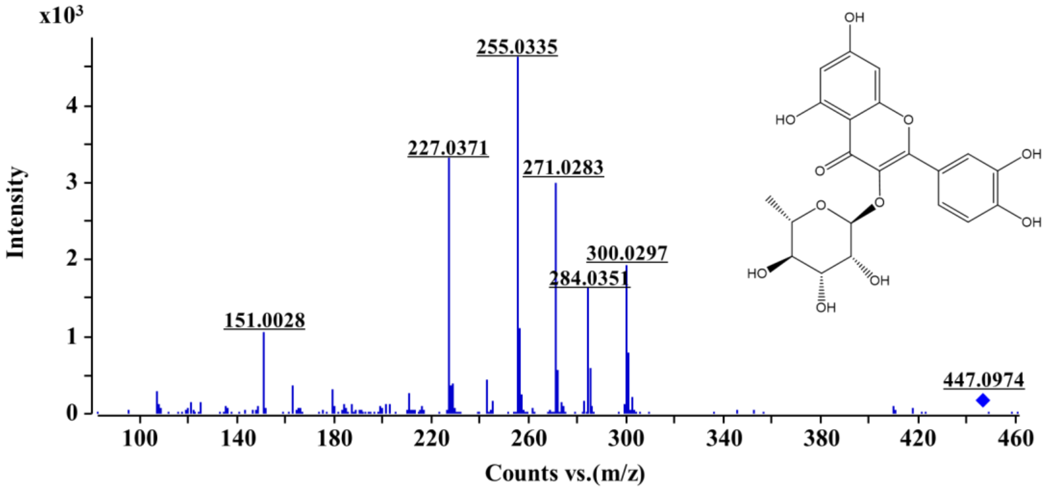

Table S1. Recovery rate and purity of the flavonoids in *Rosa setate x Rosa rugosa* wastes with different MARs.

Trade name	Purity quotient (%)	Recovery rate (%)
HPD-300	40.48 ± 0.24	83.56 ± 1.17
HP20	42.42 ± 0.24	88.13 ± 1.33

Table S2. Physical properties and manufacturer of eleven types of macroporous adsorption resins.

Trade name	Polarity	Particle diameter (mm)	Surface area (m^2/g)	Average pore diameter (nm)	Manufacturer
HP20	Non-polar	0.3-1.25	500-600	29.0-30.0	Mitsubishi Chemical Co., Ltd. (Tokyo, Japan)
HPD-100	Non-polar	0.3-1.25	650-700	8.5-9.0	
HPD-300	Non-polar	0.3-1.25	800-870	5.0-5.5	
D101	Non-polar	0.3-1.25	550-600	9.0-11.0	Cangzhou Bonchem Co., Ltd.
D312	Weak-polar	0.3-0.6	400-500	9.0-10.0	(Hebei, China)
DM130	Weak-polar	0.3-1.25	500-550	9.0-10.0	
AB-8	Weak-polar	0.2-0.6	480-520	13.0-14.0	
HZ816	Weak-polar	0.3-1.25	≥850	6.0-7.0	Shanghai Huazhen
HZ835	Middle-polar	0.3-1.25	-	-	Sci.&Tech. Co., Ltd. (Shanghai, China)
DM301	Middle-polar	0.3-1.25	330-380	14.0-17.0	Cangzhou Bonchem Co., Ltd.
HPD-500	Polar	0.3-1.25	500-550	5.5-7.5	(Hebei, China)
